# Real-Time Stylized Humanoid Behavior Control through Interaction and Synchronization

**DOI:** 10.3390/s22041457

**Published:** 2022-02-14

**Authors:** Zhiyan Cao, Tianxu Bao, Zeyu Ren, Yunxin Fan, Ken Deng, Wenchuan Jia

**Affiliations:** 1 School of Mechatronic Engineering and Automation, Shanghai University, Shanghai 200444, China; caozhiyan@shu.edu.cn (Z.C.); baotianxu@shu.edu.cn (T.B.); rzy007@shu.edu.cn (Z.R.); fanyunxin@shu.edu.cn (Y.F.); 2 Institute of Wireless Theories and Technologies Lab, Beijing University of Posts and Telecommunications, Beijing 100876, China; arieldeng@bupt.edu.cn

**Keywords:** character animation, human-robot interaction, teleoperation control, human-in-the-loop control

## Abstract

Restricted by the diversity and complexity of human behaviors, simulating a character to achieve human-level perception and motion control is still an active as well as a challenging area. We present a style-based teleoperation framework with the help of human perceptions and analyses to understand the tasks being handled and the unknown environment to control the character. In this framework, the motion optimization and body controller with center-of-mass and root virtual control (CR-VC) method are designed to achieve motion synchronization and style mimicking while maintaining the balance of the character. The motion optimization synthesizes the human high-level style features with the balance strategy to create a feasible, stylized, and stable pose for the character. The CR-VC method including the model-based torque compensation synchronizes the motion rhythm of the human and character. Without any inverse dynamics knowledge or offline preprocessing, our framework is generalized to various scenarios and robust to human behavior changes in real-time. We demonstrate the effectiveness of this framework through the teleoperation experiments with different tasks, motion styles, and operators. This study is a step toward building a human-robot interaction that uses humans to help characters understand and achieve the tasks.

## 1. Introduction

**Figure 1:**
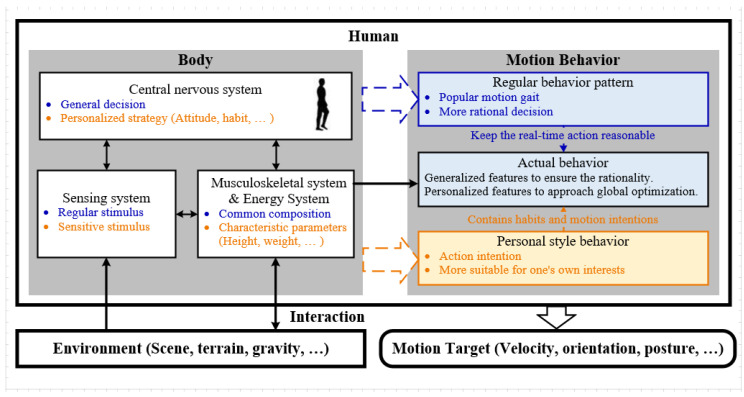
The schematic of human interaction with the environment to achieve a motion target.

In character animation and humanoid robotics, analyzing and rebuilding different interactions between humans and the environment offers the opportunity to create alternative solutions for humanoid activities. In the last two decades, research on physics-based humanoid animation and robotics improved rapidly, resulting in highly realistic and adaptive control achievements [1]. Despite this promising progress, the diversity and complexity of human behaviors, which includes a great number of behavior patterns and uncertain personal style preferences, have restricted the applications of previously proposed active controllers. The schematic of interaction based on humanoid active intention is shown in Figure 1. The human body’s central nervous system, sensing system, and musculoskeletal energy system couple together to determine behavior performance. On the one hand, people tend to make a general decision with a typical skeletal composition reacting to a regular stimulus from the environment, which therefore performs a behavior pattern, that is, for example, walking slowly after a meal. Rebuilding this pattern requires a comprehensive analysis of the environment and social habits. On the other hand, human personality styles generate tiny distinctions of this behavior pattern, such as one would like to walk in a sneaky manner while another would like to swagger down the street. These distinctions will cause an unstable disturbance for the pattern-behavior rebuilding. Not only that, but with different characteristic body parameters between humanity and controlled characters, the performance of rebuilding always becomes sensitive to the environment and unpredictable. Although plenty of works have been devoted to solving these problems, such as the SIMBICON [2] and GENBICON [3] which create simple walking controllers, the data-driven controllers [4,5] which represent modulation methods for the mocap data, the solver-based controllers [6,7] which use dynamic solvers to optimize the reference motions, and the style-based controllers [6,8] which inherit the styles of the considered movements, they could be better generalized to various scenarios and robust to diverse human behavior changes. Therefore, programming a humanoid character to achieve human-level perception and motion control in environments is still an active while challenging issue for character animation.

**Figure 2:**
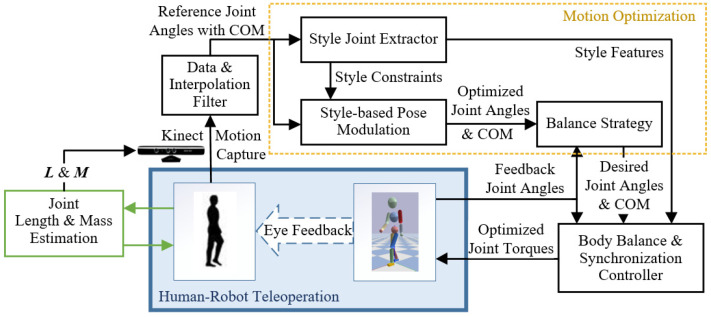
System framework overview.

Humanoid characters are complex systems that are hard to match with the sophistication and adaptability of style motion control intelligence. In addition, characters’ dexterous behavior requires high-level analysis that combines visual and proprioceptive perceptions to understand the object’s behaviors being handled. We envision humans can help the controlled characters react to these perceptions and produce whole-body style trajectories subject to these behaviors. In this work, we propose a style-based teleoperation framework to dynamically synchronize the style motion of a human operator and that of an animated character. With the help of the human operator’s perceptions and decisions to deal with different tasks in unknown environments, this framework can rebuild the human’s motion on the animated character while keeping this operator’s personal style. Besides, a human operator generates a motion with the understanding of the required task and perception of the character simulation scene, and the physics-based character then simulates a balanced motion that mimics the style of this human and achieves the task. As can be seen from Figure 2, reference motion data with the center of mass (COM) of the human body is captured as input for the data processing and motion optimization part, and an optimized motion that mimics the style of the human body is generated. The body balance and synchronization controller then computes a set of body joint torques of the optimized motion to simulate the physics-based character.

The data processing part proposes a composite scheme to smooth the visual captured human motion and COM position, and the motion optimization part optimizes the smoothed motion in consideration of the current character pose with the high-level style features. High-level features for style mimicking are extracted referring to the human-style analysis in this paper. With the high-level features, we deliver a style-expressive performance of the character keeping with the operator’s motion in real-time. The optimization is able to synthesize the balance and style to create a feasible pose under different physic scales between the human operator and simulated character. This algorithm can reduce the unstable pose error owing to the characteristic differences (such as size, preference, etc.) as well as produce stable and stylized motion data. The body balance and synchronization controller generates body joint torques, considering the balance of the character and the optimized style motion. We devise a COM and root virtual control (CR-VC) strategy, adding on the PID joint controller and design a model-based torque compensation for the CR-VC. The compensation torques modulate the joint torques to synchronize the motion, and achieve a balance and simultaneous motion control for the character.

Our system cooperates the simulated character with the human operator for the required task and, in the meanwhile, mimics the motion style of the operator. Additionally, the motion synchronization of teleoperation is achieved in this work. Our framework can be implemented online in real-time with public physic engines without any inverse dynamics’ knowledge and preprocessing requirement. No offline optimization or learning methods are needed. To our knowledge, there is currently no synchronized teleoperation system that has all these features as well as aiming to keep balance and style while achieving the task motion in real-time.

Generalization is the core advantage of our framework. Specifically, it achieves generalization across regular patterns, motion styles, and characteristic parameters. This makes our system suitable for some interactive applications such as gaming and rehabilitation. For example, a set of virtual exercises on top of this framework can be designed for the diagnosis and rehabilitation of patients with impairments [9,10,11], and this may also help increasingly immersive game variants improve user interest, enjoyment, and game-playing experience [12]. Users can interactively create desired behaviors and immediately obtain flexible, stylized, and physically simulated imitation. In this context, a wide range of results can be achieved using our method. Under the human-character synchronization, we demonstrate the generalization across different balance recovery patterns and different walking styles. They are the integration of standing, balancing, walking, and transitions between them. Additionally, through the teleoperation system, our character can navigate towards a target object placed anywhere and reach to kick it. These adapt to various fundamental motions of gaming and rehabilitation.

Our paper is organized as follows. Section 2 reviews the relevant works and Section 3 gives a human-style analysis with the system overview. In Section 4, the Kinect motion capture and data processing method is presented. The motion optimization based on the reference pose with style extractor is explained in Section 5. The body balance and synchronization controller tracking the desired pose and balancing with rhythm synchronization is explained in Section 6. In Section 7, experiments and performance details are given by a demonstration of our system under various tasks. Finally, in Section 8, we discuss this paper and mention a few ideas for future work.

## 2. Related Work

For the human-like motion animation, the physics-based controller offers an effective way to analyze the behavior patterns by visual-captured data and create the corresponding motions with balance and adaptiveness in the simulation. In recent decades, researchers worked on imitating the behavior patterns by mainly three modes as follows.

The first mode is to standardize a single regular motion pattern, and propose models and control strategies for it. Most previous works of this mode focus on walking control strategies [6,13,14,15,16,17,18] to achieve a robust, stable, and generalizable walking performance in physics-based simulation. Models are used to simplify the joint actions from the high-dimensional humanoid structure, and controllers are provided to compute the dynamics of these models. The inverted pendulum model (IPM) [13] is mainly designed for a humanoid walk, on behalf of the knee-unbent and slow speed feature of walking. Work in [14] provided a strategy to extract the IPM from the whole body to automatically adjust the desired motion and produce an adaptive walk by the velocity-driven torque method. Although it achieved effective foot placement planning, the constant length of the IPM makes it hard to be used in the humanoid multi-motion control. Many works have developed this model by adding different elements such as the linear inverted pendulum model (LIPM) [15], momentum-mapped inverted pendulum model (MMIPM) [16], double inverted pendulum model (DIPM) [17], or inverted-pendulum-based abstract model (IPAM) [18]. Although these works showed good performances of extending the walking motion to other types of motion, they are limited to performing standardized motion patterns and were hard to express the style types of one pattern. 

The second mode chooses befitting dynamic solvers to optimize the desired actions. Since humanoid actions involve high-dimensional joint groups, which will lead to complex and nonlinear dynamics of the action model, some algorithms are served to solve this problem. For example, there are the covariance matrix adaption (CMA) [19], sequential least-squares quadratic programming (SLSQP) [20], or iterative Linear Quadratic Gaussian (iLQG) [21]). Others seek to solve this problem by simplifying and pre-linearizing the complex model, such as the quadratic program (QP) in [6,7,22]. However, with a large number of samples and iterations, these methods remain computationally expensive and time-consuming for high-dimensional systems, which could cause problems for some applications that require fast and real-time responses.

The third mode adopts the kinematic balance strategy to correct the reference motion and show the feasibility by using penalty-based controllers. This mode is not limited by the characteristic simplified models, and can generate various gaits and styles of behavior patterns with low computational costs. A famous kinematic penalty-based controller is SIMBICON [2], which adopts a linear feedback-penalty framework to adjust hip joint torques directly by the stance foot position error and COM velocity error. This framework, for its intuition and high efficiency, has been widely used in researches [8,23,24,25]. Typically, the GENBICON [3] strategy developed the SIMBICON by an IPM pose design for foot placement and a transposed Jacobian virtual torque control (VTC) for COM velocity tuning. However, these works discriminately resolved the original data to partial joint trajectories and smoothed them for the pose design so that they filtered out the connotative style elements in the data. In this case, work in [8] improved the GENBICON by adding a style extractor with the COM velocity curve and step width for a walking pattern. This work has proved the effectiveness of a high-level style feature extractor for the style mimicking issue.

Our work shares some methods of these works. These methods have demonstrated the ability to perform balanced, robust, and stylized motions, like penalty-based correcting strategies [2] for joint angles and root orientation, IPM and LIPM in humanoid walking balance strategy [3,16], and some pattern features of human styles [8]. Although they have provided high anthropomorphic imitation for some motions, these works are only based on offline mocap preparation with a repeatable control strategy process. Our system does not require any offline preprocessing, nor does it rely on specific motion pattern controls. We employ real-time human motion as the mocap input and propose the teleoperation framework to improve the motion quality, and optimize it in real-time for better tracking and style expression. Previous researches that address the real-time motion imitation problem are limited. Many use wearable, similarly, measuring devices involving force/torque sensors to obtain the human motion data synchronously with different mapping methods [26,27,28,29]. Different from them, we only use the visual camera to explore a larger motion space and more free movement. Work in [30] employed the Kinect sensor and proposed a topple-free foot strategy for real-time motion reconstruction. Our framework adds a style extractor with corresponding pose optimization for a better style expression. Moreover, due to the different physic scales (size, shape, etc.) between the human operator and simulated character, we optimize the time synchronization of the system with the torque compensation based on the simplified model IPM and LIPM of the human body.

## 3. Style-Based Teleoperation Framework under Human Behavior Analysis

### 3.1. Human Style Analysis

Personal behaviors express humans’ styles of consciousness for their intentions and when finishing some tasks. Although previous works [2,3,8,20] have mentioned the “human preference and human styles” to their control strategy, they lack a complete set of human-style analyses for human behaviors. Therefore, to systematically summarize the characteristic of humans’ styles, we propose a human-style analysis in this section. We divide these styles into two parts: (1) the styles of the task motions and (2) the styles of balancing when finishing these motions. Table 1 demonstrates humanoid joints and features that represent balance and style. On the one hand, we determine there is a relatively strong correlation between the hip and shoulder joints and the active balance part, because humans tend to use these joints to recover balance when disturbed [31]. For example, when one is leaning back or forward, he prefers to wave his arms forward or back and adjust his pelvis position by the hip joint and therefore adjust the COM position [32] for balance recovery. Many hip and shoulder joint controllers [2,20] were also proposed for humanoid active balance to maintain stability among the target motion. Besides, the rest joints of the limbs are adjusted to assist the active balance for a more conformable recovery. On the other hand, expressive high-level style features are extracted from the behaviors. In this paper, we employ the foot orientation, leg motion plane, and arm motion plane as key features of expressive style inspired by [8]. This work demonstrates the style-mimicking capability of human walking, considering the swing leg plane and swing ankle trajectory. Moreover, human behavior is mainly characterized by the position and orientation of the hands and feet. The hands’ position, feet position, and COM’s heading and position are denoted as target-based style to help the human achieve the goals. Based on these analyses, we present a style-based teleoperation framework in this paper.

### 3.2. System Overview

Our style-based teleoperation framework seeks to rebuild the human’s motion on the character while keeping the motion style described above. The style elements are considered in different modules to achieve a responsive and smooth, stylized and balanced, interactive and synchronous movement task for the character. 

This framework consists of three main components, as shown in Figure 2: Kinect motion capture and data processing, motion optimization, and body balance and synchronization controller.

Kinect motion capture and data processing component captures the operator’s mocap data in real-time with 3D human body parameters’ measurement from the environment. This measurement pre-prepares the link lengths and masses of the operator’s body. The pose angles with its COM of the mocap data are then smoothed by the interpolation and filter algorithm for a smoother trajectory and transferred to the motion optimization. 

Given the smoothed data, the motion optimization component firstly annotates it with states and extracts its style features. Then the style-based pose optimization module optimizes the reference data with the constraints of style features and lower-body simple model map is improved by the famous IPM to a feasible and style pose. This modulation reduces the unstable visual recognition error and the pose error owing to the characteristic differences between human and character while maintaining the expressive and target-based style features in the motion. The balance strategy module modulates the optimized motion by current pose feedback for the balance of the character. This module mainly focuses on the hip joint modulation for an active balance style.

The body balance and synchronization controller firstly adopts a PID joint controller with gravity compensation to produce a set of joint tracking torques. Then a COM and root virtual controller (CR-VC) with model-based torque compensation is added to optimize the output torques with the active and conformable balance for the character and the target-based style synchronization for the teleoperation. The models that the torque compensation used are improved from the IPM and LIPM. Finally, the virtual physics-based character in the simulation is torque-controlled by these torques.

In a typical operation, the system works as follows. The operator generates a sequence of motions with the understanding of the required task and the simulation scene. These motions are captured by the Kinect motion capture module and transmitted to the character with joint torques. Then the physics-based character simulates a balanced motion that mimics the style of this human and successfully achieves the task. In real-time, when the simulated character is disturbed or falls into an unbalanced state, the operator adjusts his motion promptly to help the character recover into balance while proceeding with the required task.

## 4. Kinect Motion Capture and Data Processing

The sequence of the real-time reference action of the model can be obtained with a group of joint angles by Azure Kinect Body Tracking SDK [33] and the COM of the human body by 3D human body parameters’ measurement. Due to the joint difference between the Kinect model and character model, the joint angles are processed with the joint map between these two models. Then the processed joint angles and calculated COM is smoothed by the data interpolation and filter program. 

### 4.1. Joint Map between Kinect Model and Character Model

**Figure 3:**
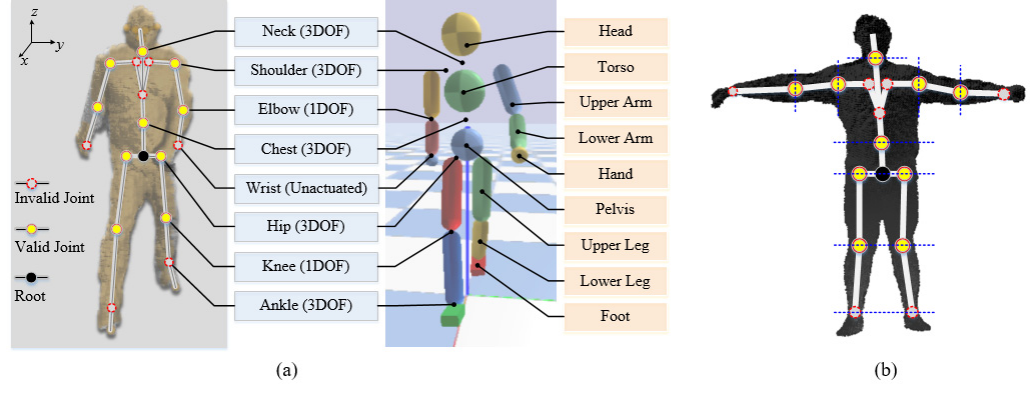
(**a**) Joint map between the Kinect model and character model; (**b**) The coronal plane projected by the point cloud of the human body. In (**a**), the diagram of Kinect tracking joints with point cloud body is on the left and that of the 3D character model is on the right. Corresponding joints with DOF are listed in the blue blocks, and links are listed in the orange boxes. In the Kinect body diagram, the valid joints for our system are marked by red-yellow circles, and invalid joints are marked by red-dotted circles. The orientations of these joints are tracked relative to their parent-link coordinate frame. The root (pelvis) link with the black circle is tracked relative to the human world coordinate to determine the human position and heading. In (**b**), the point cloud is divided into different link parts with the corresponding joints by blue lines.

The simulated character model is different from the model captured by the Kinect SDK. We adopt a 3D humanoid model for the character, which has 12 actuated joints with 13 rigid body parts linked to them. The joint angles are formed by a group of quaternion orientations for ball-and-socket joints and angles for revolute joints relative to their parent-link coordinate frame, and the position and orientation of the root link relative to the world coordinate frame. The joint map between the Kinect model and the character model is shown in Figure 3a. Some invalid joint angles (red-dotted circles) from the Kinect model are ignored due to its captured limitation [30] and some redundant degree of freedoms (DOFs) for the simulated character. Thereinto, the ankle joints are calculated by the motion optimization for their style features detailed in Section 5.3.

### 4.2. 3D Human Body Parameters’ Measurement

Due to the uneven link mass distribution of the human body, we provide the 3D human body parameters’ measurement, which contains link length and mass estimation, and COM calculation of the human body, for the Kinect motion capture. The measurement method firstly employs the joint positions and voxel point cloud of the human body. They are pre-estimated by the Kinect Body Tracking SDK as shown in Figure 3b. In this module, the length $L_{i}$ of each link i is denoted as the distance between two neighboring joint positions:

(1) $$L_{i}=\left|p_{i}^{J}-p_{i-1}^{J}\right|$$

where $p_{i}^{J}$ is the pre-estimated joint position of the link i.

Since human mass is approximately linear to the human volume with uniform density [34], the mass $m_{i}$ of link i of the human body is defined by:

(2) $$m_{i}=m_{total}*vp_{i}$$

where $m_{total}$ is the given total mass of the human body and $vp_{i}$ is the volume proportion of link i which is the ratio of the link volume to the total volume.

We provide a micro-element method that accumulates abundant micro cuboids to fit the total volume of the human body. In this point cloud, the thickness of the human body along the human sagittal axis is used as the height of the cuboid. The distance between the point at the front side $pc_{f}$ and the corresponding point at the back side $pc_{b}$ is set as the thickness of the body. We define the front side point $pc_{f}$ which can overlap the back side point $pc_{f}$ at the coronal plane. The point cloud in Figure 3b is the coronal plane section projected by the human body. The minimum thickness $t_{pc}$ between $pc_{b}$ and $pc_{f}$ is given by:

(3) $$t_{pc}=\left|pc_{f}-pc_{b}\right|$$

On this coronal plane, four neighboring points $\left[pc_{x,y},pc_{x+1,y},pc_{x,y+1},pc_{x+1,y+1}\right]$ are connected as a cross-section for a micro cuboid. We define the $pc_{x,y}$ as the position of the point at the xth row and yth column of point cloud. Therefore, the volume of this micro cuboid $v_{cub_{x,y}}$ is calculated by:

(4) $$v_{cub_{x,y}}=\left(pc_{x+1,y_{x}}-pc_{x,y_{x}}\right)\cdot \left(pc_{x,y+1_{y}}-pc_{x,y_{y}}\right)\cdot t_{pc}$$

where $pc_{x,y_{x}},pc_{x,y_{y}}$ are position on the x and y axis of the point at the xth row and yth column of point cloud, respectively.

Cuboids are traversed in the point cloud and the volume of these cuboids are summed up to calculate the whole volume of the human body $v_{total}$:

(5) $$v_{total}=\overset{n}{\underset{x,y=1}{\sum }}v_{cub_{x,y}}$$

Similarly, the volume $v_{i}$ of each link i is calculated with the corresponding part of the point cloud of the human body. The point cloud can be divided into different link parts by the corresponding joint positions. Additionally, the volume proportion $vp_{i}$ of link i is therefore determined.

Finally, the COM position $p_{COM}$ of the human body can be calculated:

(6) $$p_{COM}=\overset{n}{\underset{i=1}{\sum }}vp_{i}\cdot p_{i}^{L}$$

where $p_{i}^{L}$ is the COM position of link i recognized by Kinect SDK.

Table 2 enumerates the parameters of the links of the character model and human model and their relationships. The parameters of human model are obtained by this measurement method, and the parameters of character model are measured in the simulation. Obviously, the mass proportions of these links of the character model are different from that of the actual human model. For example, the torso mass proportion of the character model is 31.11% while that of the human model is 13.6%. Furthermore, this difference may lead the teleoperation experiment to an unpredictable motion failure. The style-based pose optimization and COM and root virtual controller in this paper can help avoid this failure, and ensure a balanced and stable movement.

### 4.3. Data Interpolation and Filter

**Figure 4:**
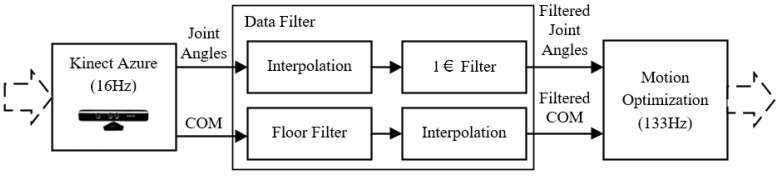
The scheme of Kinect data interpolation and filter module.

The data interpolation and filter module can smooth the obtained joint angles and COM. Figure 4 shows the details of this module strategy. Joint angles are filtered by the 1€ filter (“one Euro filter” [35]) (in this paper, named as the joint filter). The COM is filtered by the floor filter. Considering the update frequency difference between the Kinect mocap SDK and the following control loop, we use a time-varying interpolation method to avoid stepped discrete data.

The interpolation interpolates each joint angle to reach the next mocap data from the past. For example, at each step i of control loop under step n of mocap module, the interpolated angle $q_{n,i+1}^{itp}$ for next step {n,i+1} is given by:

(7) $$q_{n,i+1}^{itp}=\left(q_{n+1}^{ref}-q_{n,i}^{itp}\right)ki+q_{n,i}^{itp},\;k=\frac{f_{mcp}}{f_{itp}}$$

where $q_{n+1}^{ref}$ is the reference angle for step n+1, $q_{n,i}^{itp}$ is the interpolated angle for step {n,i}, and k is the number of interpolations, which equals the update frequency of the mocap module $f_{ref}$ over that of the control loop $f_{itp}$.

**Figure 5:**
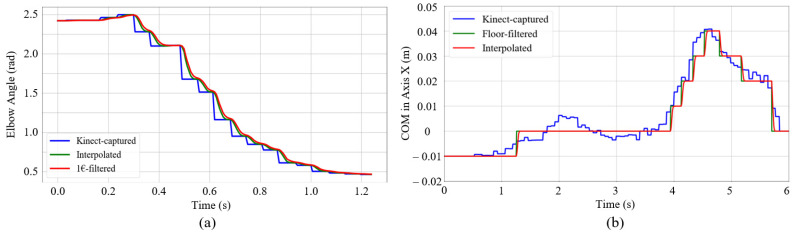
Smooth performance of the data interpolation and filter module for (**a**) the elbow angle and (**b**) the COM in *X*-axis of world coordinate. In (**a**), the Kinect-captured angle is firstly interpolated to the green line, and then joint-filtered to the red line. In (**b**), the COM value is firstly floor-filtered to the green line. and then interpolated to the red line.

Figure 5a shows the interpolation performance (green line) of the elbow angle. It yields a smooth trajectory connecting all the reference points (blue line), which ensures a smooth transition at all point corners.

The joint filter is a first-order low-pass filter with an adaptive cutoff frequency. It is suitable for the real-time Kinect joint sensor smooth because it can let the desired low-frequency human motion pass through [35] while cutting off the high-frequency signal noises with only two tuning parameters (β and $f_{cmin}$ in Equation (8)). The angle for an elbow joint of Kinect-captured data, the interpolated data, and the joint-filtered data are represented with $\left\{\beta =1,\;f_{cmin}=1\right\}$ in Figure 5a. With high computational efficiency and few tuning-process requirements, this joint filter strategy shows the responsiveness and smooth transition as the red line.

(8) $$q_{i+1}^{flt}=\left(q_{i+1}^{itp}+\frac{t}{T_{e}}q_{i}^{flt}\right)\left(\frac{1}{1+\frac{t}{T_{e}}}\right)\;t=\frac{1}{2\pi f_{C}}\;f_{C}=f_{cmin}+\beta \left|\frac{\;q_{i+1}^{flt}-q_{i}^{flt}}{T_{e}}\;\right|$$

where $q_{i+1}^{flt}$ and $q_{i}^{flt}$ are respectively the filtered reference angle for step i+1 and i, $q_{i+1}^{itp}$ is the interpolated angle from Equation (7), $T_{e}$ is the sampling period of the control loop, t is the time constant with the cutoff frequency $f_{C}$, and the intercept minimum frequency $f_{cmin}$ and the slope β are two tuning parameters detailed in [35].

Contrary to the high precision requirement of joint angle capture, the COM data capture is used for humanoid balance and thus requires relatively low precision but high stability. Therefore, we provide a floor filter for the computed COM trajectory. A threshold value is set for the floor range of the filter. Figure 5b shows the example of the COM smooth process using the floor filter with interpolation. The floor threshold is set as 0.01 m. The stable trajectory is conducive to the following adjustment for the humanoid balance in the following sections.

## 5. Motion Optimization

Although the above module has offered smoothed motion data, these data have yet to be improved to fit the character. Due to the limited accuracy of Kinect motion capture, operator behavior’s unpredictability and the characteristic differences between human and character, some motion data produced by the operator may not be suitable or even feasible for the controlled character. This section provides a motion optimization to not only make the data suitable for the current character but also inherit the style features from the operator. This optimization can be described in terms of four parts: motion states and state transitions, style feature extractor, style-based pose optimization, and balance strategy. Each of them is described in further detail.

### 5.1. Motion States and State Transitions

Humans always behave when standing with single leg or double legs. We adopt the finite state machine (FSM) [2] to annotate the reference action with three states: left stance (LS), right stance (RS), and double stance (DS). These states are defined by the desired foot-ground contact of the human operator. If the vertical height difference between the left and right foot is within a threshold error, this pose is annotated as two foot-ground contacts with the DS state. If the vertical height of the left foot is higher than the right and the difference is larger than the threshold, the pose is annotated as right foot-ground contact with the RS state. Otherwise, it is the LS state.

**Figure 6:**
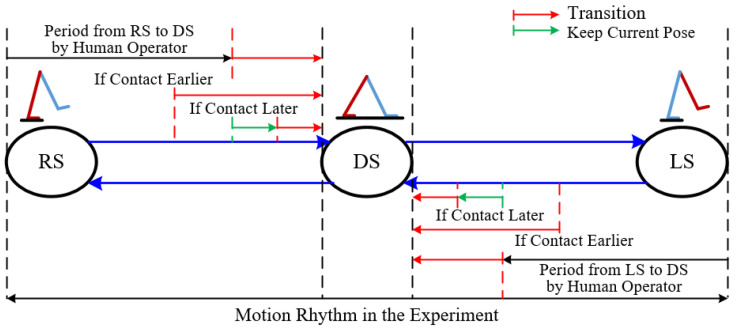
The transition between three states.

The transition between these states in Figure 6 is similar to the previous papers [2,3,4]. As in the LS or RS state of human motion rhythm, the swing foot of the character may contact the ground earlier or later than the action changes the state. If the actual contact is earlier, the transition from LS or RS state to DS state is immediately triggered. If the actual contact is later, the current reference pose is kept until the character achieves the contact and changes the state.

### 5.2. Style Feature Extractor

Based on the human style analysis in Section 3.1, high-level style features are extracted from the smoothed reference pose. The style feature extractor describes the human characteristic that constitutes the majority of both expressive and target-based style features. These features are the twist of ankles that determine the foot orientations, the leg motion plane normal curves, the relative position between the feet and COM and the heading of COM. Therefore, the style feature extractor set is denoted as $F=\left\{T_{l},\;T_{r},N_{l},N_{r},P_{cl},P_{cr},\;H\right\}$, which contains the twist angle of the left ankle $T_{l}$ and right ankle $T_{r}$ along the axis of the lower leg, the motion plane normal curves of the left leg $N_{l}$ and right leg $N_{r}$, the vector from the COM to the left ankle $P_{cl}$ and right ankle $P_{cr}$, and the heading of COM H. In this set, the ankle twist angle is extracted from the vertical axis of the ankle quaternion of the given pose. The motion plane of the leg is formed by the human upper leg and lower leg, so the normal curve of this plane should be perpendicular to the vector of both the upper leg and lower leg. The heading of COM is obtained by the vertical axis angle of the orientation of the root link.

These extracted features are delivered into the relevant modules with the following sections. Since they are extracted in the online experiment, the style feature set F is time-varying according to the real-time movement of the human body. 

### 5.3. Style-Based Pose Optimization

**Figure 7:**
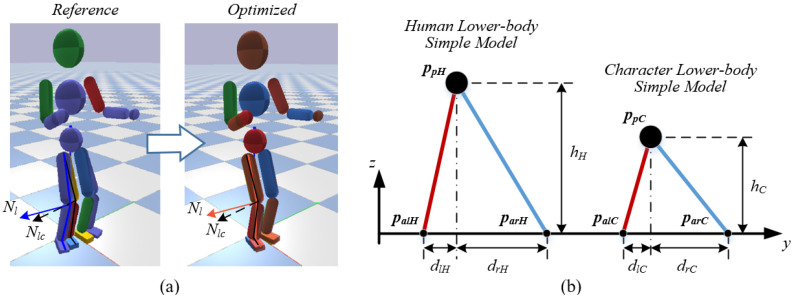
Representation of style-based pose optimization: (**a**) The generation of the optimized leg pose; (**b**) Lower body retargeting law with simplified models. In (**a**), the color model represents the current pose, the blue shadow model represents the reference pose and the red shadow model represents the optimized pose. $N_{lc}$ represents the motion plane normal curve of the left leg of the current pose and $N_{l}$ represents the motion plane normal curves of the left leg of the reference pose. The optimized pose model lands on the foot positions of the current pose with stability and inherits the leg style features from the reference pose.

As mentioned above, the real-time reference data may not be easy to follow by the character at the current pose. Directly transmitting this data to the torque controller may cause an unpredictable disturbance that leads to a stable failure for the character. It often happens when a character stands on the ground with DS state. Figure 7a indicates that the character at the current pose cannot reach the reference pose without slipping or changing its stance state. In this case, we propose the style-based pose optimization to make it feasible while maintaining the style features of this reference pose at the same time.

Firstly, the relative positions between the human ankle and pelvis links are calculated to simplify the human lower body as two connected IPM models, and the pelvis position relative to the character feet for the character’s lower-body simple model is then linearly retargeted with the human’s model. Figure 7b represents the retargeting law. The positions of the character’s left and right ankles are fixed by the current pose, and the position of the character’s pelvis is calculated accordingly. The vertical height (the z position) of a character’s pelvis $p_{pC\_z}$ is scaled by the difference between the size of human and character:

(9) $$p_{pC\_z}=h_{C}=\frac{H_{C}}{H_{H}}\cdot h_{H}$$

where $h_{C}$ and $h_{H}$ are the height of character’s and human’s lower-body, and the $H_{C}$ and $H_{H}$ are the given height of character and human model, respectively.

The horizontal position (the x and y position) of the pelvis $p_{pC}$ relative to the left ankle $p_{alC}$ and right ankle $p_{arC}$ is defined by the relationship between $d_{lH}$ and $d_{rH}$:

(10) $$\{\begin{array}{c} p_{pC\_x}=p_{alC\_x}+\left(p_{arC\_x}-p_{alC\_x}\right)\cdot \frac{d_{lH\_x}}{d_{rH\_x}}\\ p_{pC\_y}=p_{alC\_y}+\left(p_{arC\_y}-p_{alC\_y}\right)\cdot \frac{d_{lH\_y}}{d_{rH\_y}} \end{array}$$

Once the character’s simple model is determined, it becomes a three-dimensional inverse kinematics (IK) problem to calculate the optimized hip and knee orientation, which has an infinite number of solutions. As mentioned by study [8], this problem can be constrained to a unique solution by specifying the signed inverse kinematics plane. We employ the plane normal curves by the leg motion plane normal curves $N\in \left\{N_{l},N_{r}\right\}$ of features set to determine the inverse kinematics plane. It converges the three-dimensional IK problem to a two-dimensional IK problem with one unique solution. Figure 7a displays the optimization process of the hip and knee orientation of the character. The reference pose in blue shadow is optimized to the red shadow pose, which shows the operability and style inheritance.

Lastly, we calculate the target foot heading with the foot style feature $T\in \left\{T_{l},\;T_{r}\right\}$. Generally, the contact foot should be smoothly parallel to the ground for the full foot landing requirement. After the foot orientation is decided, the ankle orientation is accordingly computed by the quaternion difference between the lower body orientation and foot orientation.

### 5.4. Balance Strategy

Human balance behavior heavily relies on the root orientation and hip joints. We adopt the SIMBICON [2,4] modulation law to the root and hip joints.

In our system, for the balance of character motion, it is better to keep the transverse plane of the character body parallel to the ground. Therefore, the root link orientation in the X and Y axis is defined parallel to the X-Y plane of the world coordinate, and the root link orientation in the Z-axis equals the heading of COM {H}.

The hip joints are also modulated according to the character motion. In the LS and RS state, for 3 DOF stance hip joints, the desired angle $q_{d\_sth}$ is modulated by the reference stance hip joint $q_{r\_sth}$, and the current stance hip joint $q_{c\_sth}$ with the weight $w_{0}$:

(11) $$q_{d\_sth}=q_{r\_sth}^{w_{0}}\cdot q_{c\_sth}^{1-w_{0}}$$

For each DOF swing hip joint, the balance modulation is applied to desired swing hip joint angle $q_{d\_sw}$ as:

(12) $$\theta_{d\_sw}=\theta_{r\_sw}+c_{d}\left(d_{d}-d_{f}\right)+c_{v}\left(v_{d}-v_{f}\right)$$

where $\theta_{r\_sw}$ is the reference swing hip joint angle, $d_{d}$ is the desired horizontal movement distance, $d_{f}$ is the horizontal feedback movement distance from simulation, $v_{d}$ and $v_{f}$ are the desired and feedback horizontal velocity of COM, and $c_{d}$ and $c_{v}$ are the gain parameters referring to [2], respectively.

## 6. Body Balance and Synchronization Controller

**Figure 8:**
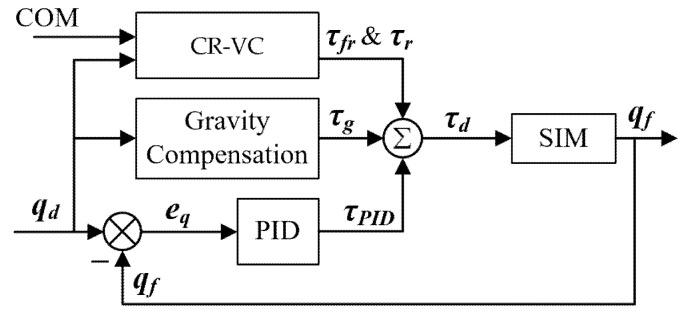
The scheme of the body balance and synchronization controller.

The physics-based character in the simulation is controlled by the joint torques. This component computes the desired joint torques to keep the motion balance and synchronization for the character. The control scheme is detailed in Figure 8. A PID joint controller with gravity compensation is adopted for the desired joint tracking performance. And a COM and root virtual control (CR-VC) strategy with model-based torque compensation is proposed for torque optimization. This strategy maintains the active and conformable balance by adjusting the corresponding joint torques. The model-based torque compensation employs the target-based style features of building models and compensates the torques, keeping the time synchronization of the system.

### 6.1. PID Joint Controller with Gravity Compensation

A close-loop discrete PID joint tracking controller is implemented with gravity compensation. For each joint DOF at each timestep of simulation, the desired joint torque is tuned with angle position feedback to enable accurate tracking performance. A discrete controller is implemented with a PID algorithm by:

(13) $$\begin{array}{cc} \tau_{PID}\left(t+1\right)= & K_{p}\left[q_{d}\left(t+1\right)-q_{f}\left(t\right)\right]+K_{i}\overset{t}{\underset{1}{\sum }}\left[q_{d}\left(t+1\right)-q_{f}\left(t\right)\right]\\ & +K_{d}\left\{\left[q_{d}\left(t+1\right)-q_{f}\left(t\right)\right]-\left[q_{d}\left(t\right)-q_{f}\left(t-1\right)\right]\right\} \end{array}$$

where $\tau_{PID}\;$is the generated desired joint torque, $q_{d}\;$is the desired joint angle from Section 5, $q_{f}$ is the actual joint angle feedback in the simulation, and $K_{p},\;K_{i},\;K_{d}$ are the PID gains, respectively.

Since the tracking performance of the controller will be influenced by the humanoid’s gravity, a gravity compensation module is added to pre-compute the compensation torque to the motion tracking controller. We adopt a transposed Jacobian method [3], which employs the virtual inverse gravity force as compensation. For each link i, the virtual force $f_{i}=-m_{i}g$ is applied to the COM of the link. Then the $f_{i}$ is transposed to the torques $\tau_{gi}$ by:

(14) $$\tau_{gi}=J_{i}^{T}f_{i}$$

where $J_{i}^{T}$ is the Jacobian of the COM for a chain of links from the root link to the link i with respect to joint angle $q_{d}$, thus $\tau_{gi}$ is computed as a group of torques applied to the joints between the root link and the link i.

To sum up the virtual force of all links, the total compensation force is:

(15) $$\tau_{g}=\overset{n}{\underset{i=1}{\sum }}J_{i}^{T}f_{i}$$

### 6.2. COM and Root Virtual Controller (CR-VC)

Generally, researchers [2,3,4,5] control the root position and orientation to ensure the robustness and balance of the high-dimensional character that has a “floating base” [36]. In this paper, the character base is defined as the pelvis link. We propose a COM and root virtual control (CR-VC) strategy for the balance of the character. Thereinto, the COM virtual force controls the position of the pelvis and the root virtual torque controls the orientation of the pelvis. Both virtual force $f_{r}$ and torque $\tau_{r}$ are computed by the PD control strategy as Equation (16). Since the root of the character is a floating base, the virtual torque can only be realized using internal torques [2] and distributed to the stance hips of the character:

(16) $$\{\begin{array}{c} f_{r}=Kp_{vf}\cdot e_{p_{r}}+Kd_{vf}\cdot e_{{\dot{p}}_{r}}\\ \tau_{r}=Kp_{vt}\cdot e_{q_{r}}+Kd_{vt}\cdot e_{{\dot{q}}_{r}} \end{array}$$

where $e_{p_{r}}$ and $e_{{\dot{p}}_{r}}$ are the position and position velocity error represented in the following section, $e_{q_{r}}$ and $e_{{\dot{q}}_{r}}$ are the orientation and orientation velocity error of the pelvis, and $Kp_{vf}$, $Kd_{vf}$, $Kp_{vt}$, and $Kd_{vt}$ are the PD gains for the virtual force and virtual torque, respectively.

The virtual force $f_{r}$ is transposed into the corresponding joint torques, similar to Equation (14):

(17) $$\tau_{fr}=J_{r}^{T}f_{r}$$

where $J_{r}^{T}$ is the Jacobian of the COM for a chain of links from the pelvis to the stance foot, thus $\tau_{fr}$ is computed as a group of torques applied to the joints between the root link and the stance foot.

In conclusion, the set of the body balance and synchronization control torques for the character is:

(18) $$\tau_{d}=\tau_{PID}+\tau_{g}+\tau_{r}+\tau_{fr}$$

### 6.3. Model-Based Torque Compensation for the CR-VC

**Figure 9:**
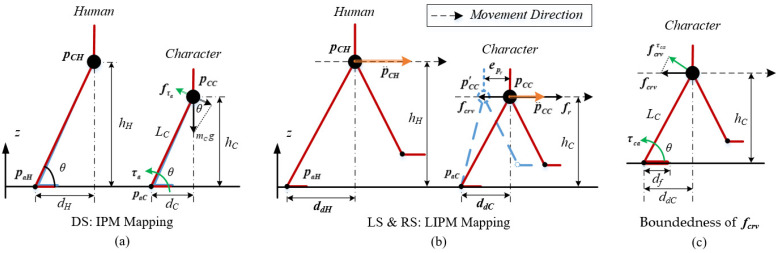
(**a**) The map of the IPM between the human operator and character; (**b**) The map of the LIPM between the human operator and character; (**c**) The boundedness representation of $f_{crv}$.

Due to the high-dimensional humanoid structure, the computation of the character model is needed to be simplified for the CR-VC method. Since humans often double-stand under a quasi-static state and walk parallel to the ground, we adopt the famous IPM to simplify the human DS motions and LIPM for human RS and LS motions. Figure 9a,b shows the maps of the IPM and LIPM model between the human operator and character. Both IPM and LIPM are composed of a point mass and a massless segment connecting this point mass to a ground contact point. The massless segment is generated by the target-based style vector $P_{c}\in \left\{P_{cl},P_{cr}\right\}$ from the style feature extractor set. The virtual force of the CR-VC method with the position error $e_{p_{r}}$ and velocity error $e_{{\dot{p}}_{r}}$ of the character’s COM is calculated with these models:

(19) $$\{\begin{array}{c} e_{p_{r}}=p_{CC}-p_{CC}^{\prime }\\ e_{{\dot{p}}_{r}}={\dot{p}}_{CC}-{\dot{p}}_{CC}^{\prime } \end{array}$$

where $p_{CC}$ is the desired and measured COM position of the character model scaled by human’s COM position, $p_{CC}^{\prime }$ is the measured COM position of the character model, ${\dot{p}}_{CC}$ and ${\dot{p}}_{CC}^{\prime }$ are the corresponding velocities, respectively.

The mathematical details of the IPM and LIPM have been provided by many previous works [13,15]. Different from them, we added an ankle link at the bottom of each model, because sometimes it is necessary to consider the ankle torque for maintaining the balance of humans. The IPM in Figure 9a indicates the necessity. When the operator stands, he or she may not be exactly vertical to the ground. Therefore, the reference standing angle θ may not be 90° and a rotation component force produced by gravity occurs. To compensate for this force, an ankle torque $\tau_{am}$ is added to the controller:

(20) $$\tau_{am}=m_{C}g\cos\theta L_{C}=m_{C}gd_{C}$$

where $m_{C}$ is the total mass of the character and $d_{C}$ is the horizontal distance of the natural length $L_{C}$ of the IPM.

Besides, compensation for the virtual force of the CR-VC method is also needed on account of the unstable nature of the LIPM. As mentioned above, the physical scales of character and human operator are always different from each other. The linear natural frequencies of the LIPM of them are also different. This will cause different motion rhythms when the operator controls the character and disturbs the teleoperation [15]. To unify the rhythms of different models with different physical scales, the time synchronizing compensation for LIPM mapping is proposed. 

In Figure 9b, the equation of motion for the LIPM is given by:

(21) $$p_{ai}=p_{Ci}-\frac{{\ddot{p}}_{Ci}}{w_{i}^{2}},\;i=C,H$$

where $p_{Ci}$ is the horizontal position of COM, $p_{ai}$ is the horizontal position of the center of pressure (CoP), $w_{i}=\sqrt{g/h_{i}}$ is the linear natural frequency of the LIPM, and i indicates the character or the human model. In the LS and RS state, the CoP is defined as the horizontal position of the ankle joint, and $w_{i}$ governs the characteristic time response of this model.

Previous works have studied that if two motions can be made identical by multiplying all lengths by a ratio and all forces by the same ratio [37], they are defined as dynamically similar. It requires both the operator and character to match their COM positions as well as their first- and second-order derivative simultaneously [15]. Due to the unactuated characteristic of LIPM, matching these may generate infeasible trajectories with different natural frequencies. For example, when $\frac{p_{CH}}{h_{H}}=\frac{p_{CC}}{h_{C}}$, LIPM mapping law produces ${\ddot{p}}_{CH}={\ddot{p}}_{CC}$ by Equation (21). However, it should be $\frac{{\dot{p}}_{CH}}{h_{H}}=\frac{{\dot{p}}_{CC}}{h_{C}}$ and $\frac{{\ddot{p}}_{CH}}{h_{H}}=\frac{{\ddot{p}}_{CC}}{h_{C}}$ to achieve the synchronization of the mapping motion.

To achieve the match, we add a root virtual force $f_{crv}$ for the CR-VC to minimize the difference between the desired acceleration ${\ddot{p}}_{CC}=\frac{{\ddot{p}}_{CH}}{h_{H}}\cdot h_{C}$ and the actual acceleration ${\ddot{p}}_{CC}={\ddot{p}}_{CH}$.

(22) $$f_{crv}=m_{C}\left(\frac{{\ddot{p}}_{CH}}{h_{H}}\cdot h_{C}-{\ddot{p}}_{CC}\right)$$

Substituting Equation (21) into (22):

(23) $$f_{crv}=m_{C}g\left(\frac{p_{CH}-p_{aH}}{h_{H}}\cdot \frac{h_{C}}{h_{H}}-\frac{p_{CC}-p_{aC}}{h_{C}}\right)=m_{C}g\left(\frac{d_{dH}}{h_{H}}\cdot \frac{h_{C}}{h_{H}}-\frac{d_{dC}}{h_{C}}\right)$$

### 6.4. Movement Boundedness of the CR-VC

Due to the limited length and width of the footplate, there exists movement boundedness for the CR-VC method in our system.

It is intuitive that for the DS state, the projection of $p_{CC}$ on the ground should be located in the standing area. Therefore, the tilting horizontal distance $d_{C}$ of IPM should be less than the minimum of the length and width of the footplate:

(24) $$d_{C}<\min\left(L_{f},\;W_{f}\right)$$

Besides, in LIPM, the rotation component force $f_{crv}^{\tau_{ca}}=f_{crv}\sin\theta$ is produced from the virtual compensation force $f_{crv}$. This component force is perpendicular to the link $L_{C}$, and is actually applied to the ankle joint according to Figure 9c. The corresponding ankle torque $\tau_{ca}$ is generated by Equation (25):

(25) $$\tau_{ca}=f_{crv}^{\tau_{ca}}L_{C}=f_{crv}h_{C}$$

However, to avoid foot flipping, the ankle compensation torque should be less than the footplate flip force $\tau_{flip}$:

(26) $$\tau_{ca}<\tau_{flip}$$

(27) $$\tau_{flip}=m_{c}gd_{f},d_{f}=\min\left(L_{f},\;W_{f}\right).$$

Substituting Equations (23) and (27) into (26), the walking distance $d_{dC}$ of the character is calculated as bounded with respect to the human-character size scale:

(28) $$d_{dC}<\frac{d_{f}}{\frac{h_{C}}{h_{H}}-1}$$

## 7. Experimental Results

### 7.1. Experimental Platform

**Figure 10:**
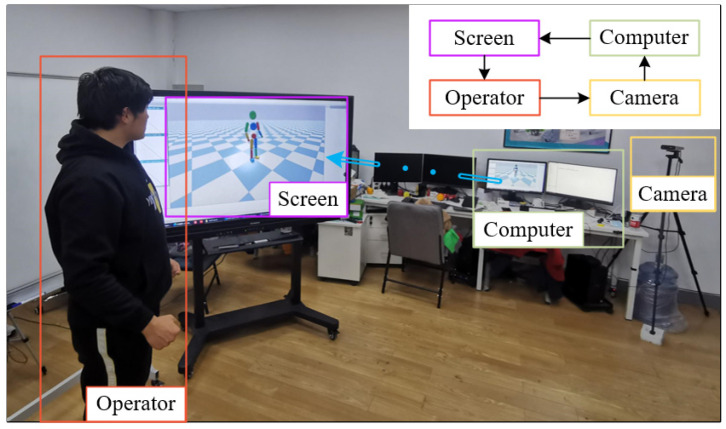
The teleoperation experimental platform.

The effectiveness of our teleoperation framework is demonstrated with the experimental platform in Figure 10. The operator in the platform generates the motions with the understanding of the required task and the reaction to the simulation environment on the screen. The computer captures the human motion data from the Azure Kinect camera and produces the joint actuating torques for the character by this data. Then the performance of the controlled character is displayed on the screen and as visual feedback for the operator. This platform validates the human-character motion teleoperation performance via visual feedback and the framework controller. 

All simulations were run on a computer with an Intel Core i5-8600K CPU, 16G memory. The PyBullet physics engine [38] is used for the forward simulation and supplied as visual feedback with the gravity constant $G=9.8\;m/s^{2}$ and integration time step 1/600 s. The ground reaction is modeled as a damped spring with spring coefficient 30,000 N/m and damping coefficient 10,000 Ns/m and friction coefficient 0.9. The update frequency of the Kinect motion capture is 16 Hz, and the update frequency of the following control loop is 133 Hz. The mass of the character model is 45 kg, and the height is 1.62 m. The link attributes of the operator and character are listed in Table 2.

### 7.2. Parameter Settings

In this paper, the PID feedback gains are manually tuned with the fixed root link. We repeat this tuning procedure for all DOFs of the joints. (1) Ramp responses are considered to test PID gains in the range of the operating angles. The slope of the ramp function for each joint is set as the maximum slope value of the reference trajectory of this joint. (2) Samples of PID are tested and the total position error of the tracking performance is summed. The corresponding PIDs with the minimum error are selected as the suitable value. The parameter results are displayed in Table 3. The PID gains of the upper body joints are approximately related to the mass of the corresponding links while the gains of lower body joints achieve stable tracking performance. The $c_{d}$ and $c_{v}$ are relatively small in our system. To better demonstrate the applicability of our teleoperation system, we set $c_{d}$ and $c_{v}$ equals to zero in the following experiments.

Another gain parameter set is for the CR-VC method. The CR-VC parameters are tuned to minimize the COM difference between the operator and the character when they double-stand on the ground. Since the stability of root orientation is much more important than others, the gains for the virtual torque are set higher to reach a stronger control. These parameters are only tuned once at the beginning. Under a feasible range of motion behaviors, our framework is not particularly sensitive to these values. With different motion behaviors and different human operators, the following experiments all adopt this set of parameters and successfully finish the tasks.

### 7.3. Remarks for Teleoperators

Before the experiment, there are a few remarks that should be kept in mind for the operator when applying the teleoperation. These are as follows.

1. The motion data captured by the Kinect camera should be unabridged, so the operator should act more than two meters away from the camera for the whole-body motion capture.
2. While walking, the operator’s lower body needs to be kept bent to satisfy the LIPM simplification of the pose optimization and the feasibility of its IK solution.
3. When the operator double-stands on the ground, the COM should be landed in the standing area to avoid foot flipping.
4. The stepping length of the operator is bounded by Equation (28).
5. High dynamic motions, of which models conflict with IPM or LIPM, should be avoided.

### 7.4. Experiments

To validate the proposed framework, we analyze the system performance when the human operator interacts with the simulated character. First, we evaluate the human-character synchronization performance with the CR-VC torque compensation. Next, we show how the operator manipulates the character, and the character follows to achieve different tasks with patterns and styles. Finally, we display the generalization of our system with different operators and some model-unsuitable motions.

#### 7.4.1. Teleoperating under Human-Character Synchronization

**Figure 11:**
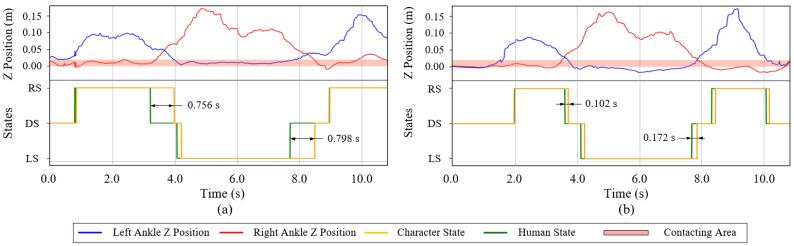
The comparison of the stepping situation between the CR-VC (**a**) without torque compensation and (**b**) within torque compensation.

We evaluate the synchronization performance of our system in this section. Due to the size, shape, and link attribute differences between the operator and character, the problem in Section 6.3 may occur without the CR-VC torque compensation in the teleoperation. Figure 11a represents the uncompensated situation. When the operator treads down his left foot (blue line) and the human state transits from the RS state to the DS state (green line), the character’s COM and the state transition (yellow line) could not catch up with the human’s in time due to the physical difference. This delays up to 0.798 s and causes a huge disturbance for the following control. Through the experiment, the character falls within 3 steps. Whereas, in Figure 11b, the compensation torques adjust the CR-VC torques calculated in real-time, the character’s COM can follow the human’s COM and the maximum delay of the state transition is lower than 0.172 s. Since the human visual processing response time is approximately 200~250 ms [39], the system with this delay can achieve the task teleoperation.

#### 7.4.2. Teleoperating with Different Patterns

A distinct advantage of our system is that it allows free motions reacting to the task and environment, which are dependent on the operator’s personal wills. The comprehensive analysis is generated by the operator and the simulated character only needs to follow the operator’s motion to achieve his will. We demonstrate this advantage with a balance recovery experiment in comparison with previous works. Although the robustness of the simple balance strategy has been proved in [1,2,3,8], their balance recovery performance heavily relies on the current state of character motion. Most of them require the character to be at the continuously stepping state when being pushed. However, our character can bear the interrupt push at any time state, including the standing state. Moreover, it can act with different patterns to recover the balance according to the current disturbing situation. 

**Figure 12:**
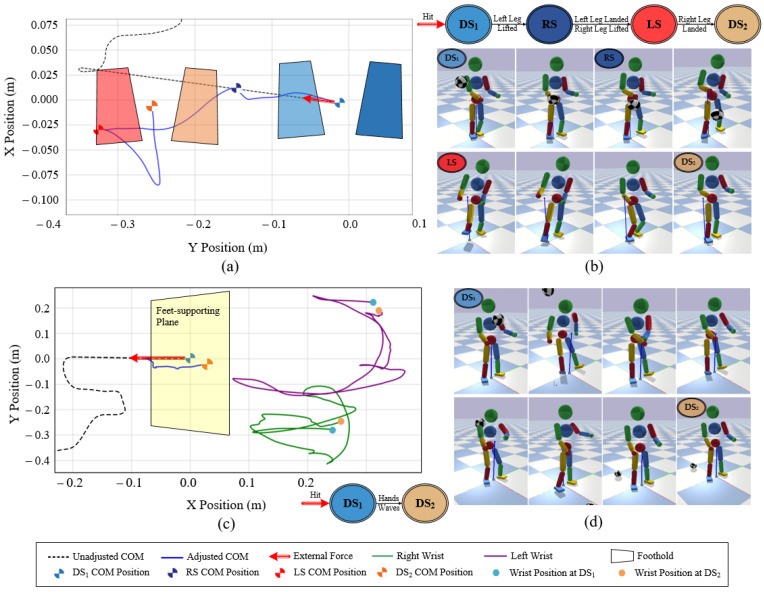
Recovery experiments with two patterns responding to the ball push: (**a**) The COM adjusting trajectory (blue line) within 4 steps. Four steps are the light blue trapezoids at the first double stance state DS1, the deep blue trapezoid at the right stance state RS, the light red trapezoid at the left stance state LS, and the orange trapezoids at the second double stance state DS2; (**b**) Snapshots of the balance recovery process by initiatively stepping; (**c**) The COM adjusting trajectory (blue line) with the trajectories of the left hand (purple line) and right hand (green line); (**d**) Snapshots of the balance recovery process by waving the hands.

Figure 12 shows two recovery patterns responding to the current disturbance. When the character stands on the ground with double legs, it gets hit by a football and tends to fall. The operator in real-time moves himself to manipulate the character return to balance. The operator in Figure 12a,b controls the character returning to the normal stand by lifting its leg to adjust its COM. In detail, when the operator sees the ball coming and hitting the character, he initiatively lifts his left leg and adjusts the leg landing position with the observation of the character’s pose. The system in real-time changes the stance state of the character and actuates it to follow the motion of the operator. When the character successfully steps his left foot, the operator then lifts his right leg to achieve a more stable standing pose for the character. The COM adjusting trajectory of this recovery experiment is drawn in Figure 12a, and the state transitions and the sequential shots of the system operation are given in Figure 12b. Figure 12c,d displays another recovery strategy pattern, that is, waving its hands for the character to recover balance. When the ball hits the character in the front direction, the character tends to fall back. At this time, the operator waves his hands. The character is actuated following the operator’s pose and successfully holds its body as a result. Figure 12c draws the COM of the character adjusted back to the feet-supporting plane with stability. Results of these tasks show the robustness of our teleoperation system and it can adapt to different scenarios by different recovery motion patterns. Moreover, the state transition can be controlled by the operator to achieve the stepping recovery strategy without transition preprocessing, which is another advantage of our system.

#### 7.4.3. Teleoperating with Different Styles

**Figure 13:**
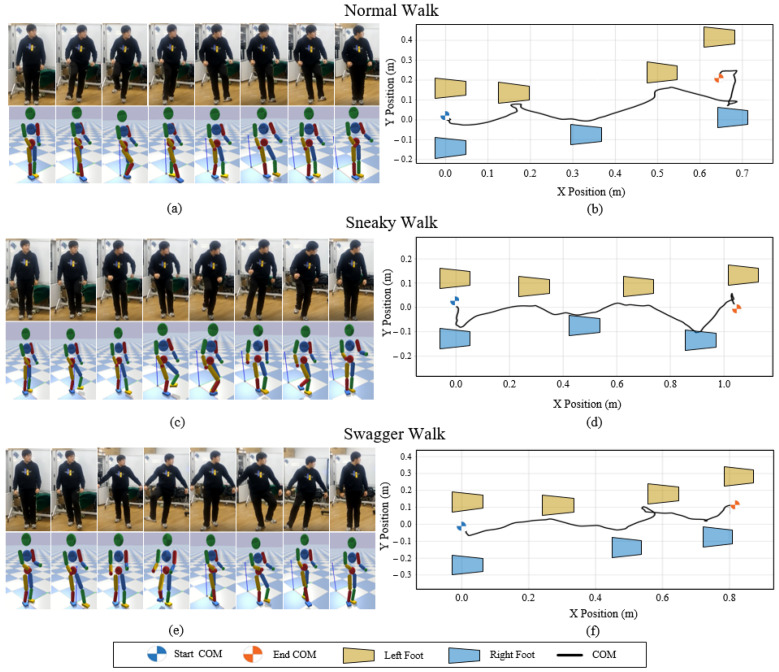
Walking experiments with different styles: normal, sneaky, and swagger. (**a**,**c**,**e**) the snapshots of the normal walk, sneaky walk, and swagger walk teleoperation process with the operator and the character, respectively. (**b**,**d**,**f**) the COM trajectory of the character with footsteps.

**Figure 14:**
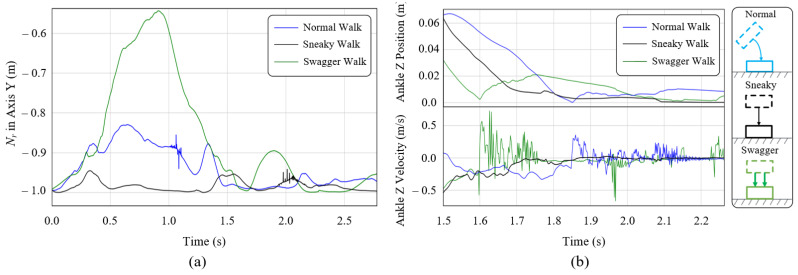
(**a**) The Y-axis component of the motion plane normal curves of the right leg $N_{r}$ of different walking styles in the world coordinate. (**b**) The Z-axis components of the right ankle positions and velocities in different walking styles.

To demonstrate the style mimicking capability of our teleoperation system, we design the walking experiments with different walking styles by the operator. They are normal walk, sneaky walk, and swagger walk. The results of these experiments reveal that our system is capable of mimicking different styles of the operator’s motions in real-time. In the experiments, the operator moves with the understanding of the required walking motion and the simulation scene, and the character follows the motions captured by the operator. The sequential shots of the teleoperation in Figure 13a,c,e show the teleoperating processes and the COM trajectories are drawn in Figure 13b,d,f, respectively. These results indicate that our system satisfies a compromise between keeping balance and mimicking different walking styles in real-time. Figure 14a draws the horizontal component of motion plane normal curves of the right leg $N_{r}$ in the character’s walking motion with different styles. The swagger motion displays larger horizontal change and the sneaky motion displays lower. The corresponding ankle heights in Figure 14b represent larger stepping impact with larger stepping velocity vibration in swagger walk (green line), and lower stepping impact with lower stepping velocity vibration in sneaky walk (black line).

The COM trajectory is very important in analyzing the walking motions. Figures show that before the walk, the COM tends to move horizontally away from the swing side as preparation so that the swing leg can leave the ground. Furthermore, through the walking process, the COM moves between the legs. Interestingly, the swagger motion produces a larger facing wagging but less COM side-to-side moving distance, while the normal motion produces a larger movement. We infer that, in a comfortable normal walk, humans tend to walk with fewer energy costs. They may walk more like an IPM’s switching process when alternating their standing legs rather than the LIPM’s switching process, which may cause a larger side-to-side movement distance.

#### 7.4.4. Teleoperating for a Target

**Figure 15:**
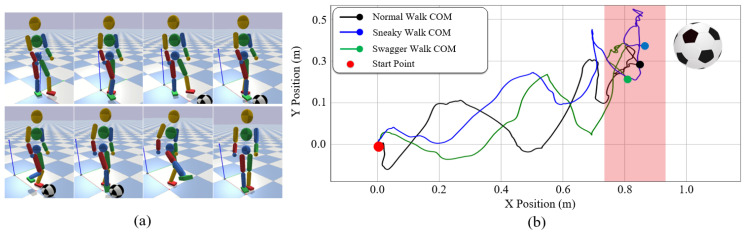
(**a**) Snapshots of the football kicking process with the sneaky walk; (**b**) The COM trajectories of the character with three walking styles to kick the football.

We also design a target-based teleoperation task to demonstrate the usage of the target-based style features in our system. In this experiment, the operator is required to walk forward to the front of a football and kick it with the above three walking styles. Figure 15b shows the ability of the character to kick the ball in these walks. Through the teleoperation, the character successfully reaches the feasible kicking area (red shadow) and, after adjusting its COM for a while to keep the balance, kicks the football.

#### 7.4.5. Generalization of the Teleoperation

**Figure 16:**
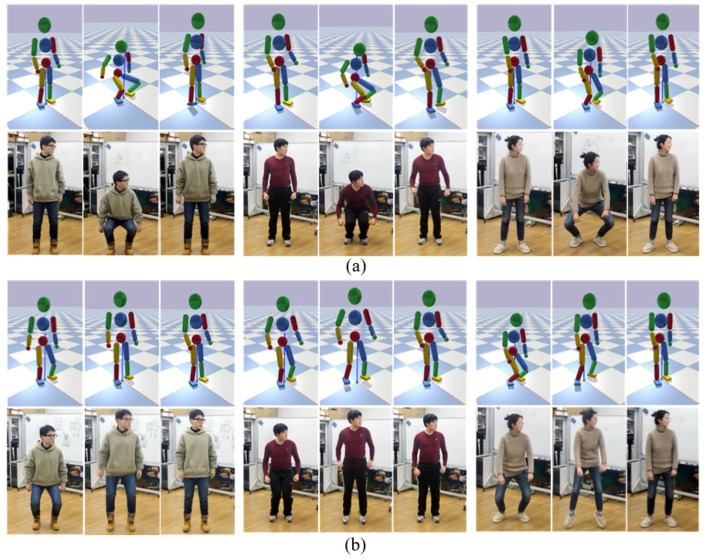
(**a**) Snapshots of the squat motion by different operators; (**b**) Snapshots of the jump motion by different operators.

Our teleoperation system can generalize across different tasks and different operators and are able to interact with them. To demonstrate the generalization, in addition to the walking and ball kicking motions mentioned above, we also test some tasks of which the motion cannot be simplified by the corresponding model in this paper with different human operators. The character controlled by our system can follow different operators with different sizes and shapes to achieve the squat motion and jump motion, as shown in Figure 16. After the squat and jump position, it can stand firmly back to the DS state.

## 8. Discussion

We have presented a style-based teleoperation framework for motion synchronization and style mimicking to achieve the tasks within the human-character interactions in real-time. In this work, the human operator can help the character understand the required tasks and percept the environment, and the character can achieve the tasks by following the human motion while maintaining the human style and keeping adaptable balance. The resulting motions are robust under external disturbances by different balance recovery patterns, like changing the stance states or waving the hands, decided by the human operator. Additionally, it can mimic styles under distinct style differences with task achievement and avoid the balance failure caused by the characteristic difference between the operator and character. Our framework does not rely on inverse dynamics or dynamic solvers for computation and can run in the publicly available forward simulation engines without any preprocessing of offline optimization or learning methods. These features make our method useful for many interactive applications such as games and rehabilitations. We expect this system to amplify a user’s authoring effort in creating more comprehensive behaviors based on the environment and social habits. It can be a circular chain formed by the human secular cognition and the physics-based character animation alternatively, and can promote the interaction and integration between them for a more anthropomorphic virtual world.

Certain limitations still exist. There are some limitations of the operator motions in our system. Due to the model-based torque compensation in Section 6.3, the operator motions should fit the simplified model like the IPM in the DS state and LIPM in the LS and RS state. This restricts the motions from high dynamic or abnormal movements. Another drawback of our method is that it does not guarantee the motion feasibility of the character. Our system partially relies on human perception and understanding of the current tasks and situations. If the human is unresponsive or unskilled in operating the character, the control performance may not be as good as it should be. Future work may involve employing a pattern and style database to predict the next pose with respect to the required tasks and human pose. This can help humans control the character easier. The integration of some strategies for solving high-dynamic motions with high computational efficiency should be another interesting future direction. This would provide the users to create rich styles and patterns of motion in the teleoperation. Moreover, with rich styles and patterns, multi-person situations can be considered to create a more socialized and comprehensive strategy.

## Figures and Tables

**Table 1:** Human preference for joint and link usages.

Balance		Style	
Hip	Active Balance	Feet orientation	Expressive Style
Shoulder		Leg Motion Plane	
Knee	Conformable Balance	Arm Motion Plane	
Elbow		Hands’ and Feet Position	Target-based Style
Ankle		COM Heading and Position	
Wrist			

**Table 2:** Link parameters of the character and human model.

Link Name	Character Link Attributes			Human Link Attributes		
	Mass (kg)	Size (m)		Mass (kg)	Size (m)	
Pelvis	6.0	Sphere Radius	0.09	19.8	Radius	0.17
Torso	14.0	Sphere Radius	0.11	10.3	Radius	0.15
Head	2.0	Sphere Radius	0.10	5.7	Radius	0.09
Upper Arm	1.5	Radius	0.05	2.0	Radius	0.08
		Length	0.18		Length	0.20
Lower Arm	1.0	Radius	0.1	1.2	Radius	0.1
		Length	0.14		Length	0.20
Upper Leg	4.5	Radius	0.06	11.5	Radius	0.09
		Length	0.30		Length	0.40
Lower Leg	3.0	Radius	0.06	3.3	Radius	0.40
		Length	0.31		Length	0.40
Foot	1.0	Length	0.18	2.0	Length	0.30
		Width	0.08		Width	0.10
		Height	0.05		Height	0.06

**Table 3:** Gain parameters of our system.

**Joint Name**	**Gain Parameters of PID Controller**		
	$K_{p}$	$K_{i}$	$K_{d}$
Chest	2500	40	36
Neck	30	5	3
Hip	500	100	20
Knee	360	72	20
Ankle	60	15	2
Shoulder	60	15	7
Elbow	60	15	1
**Virtual Compensation**	**Gain Parameters of CR-VC**		
	** $K_{p}$ **	** $K_{p}$ **	
Virtual Force	50	30	
Virtual Torque	5000	1000	

## Data Availability

Not applicable.
